# Frontiers of inflammatory disease research: inflammation in cardiovascular–cerebral diseases

**DOI:** 10.1186/s41232-021-00160-z

**Published:** 2021-03-29

**Authors:** Daiju Fukuda, Masataka Sata

**Affiliations:** Department of Cardiovascular Medicine, Tokushima University Graduate School of Biomedical Sciences, 3-18-15, Kuramoto-Cho, Tokushima, 770-8503 Japan

Inflammation is defined as a defensive reaction involving the immune system. However, chronic inflammation induced by prolonged stresses, including infection, physiological stress, or overnutrition, activates immune cells and/or interstitial cells, causing tissue remodeling, which plays a pivotal role in the pathogenesis of chronic inflammatory diseases. Chronic inflammation is associated with atherosclerotic vascular diseases, heart failure, metabolic syndrome, neurodegenerative diseases, cancer, and others.

For example, Virchow had already reported that atherosclerosis is an inflammatory disease in the nineteenth century [1]. However, currently, only a few therapeutic strategies targeting inflammation have evidence for atherosclerosis prevention. The major medical science goal is to develop effective, well-refined therapies. Accomplishing this task requires the continuation of research and a comprehensive understanding of the pathophysiology of these diseases. In this thematic series, Frontiers of inflammatory disease research: Inflammation in cardiovascular–cerebral diseases, in *Inflammation and Regeneration*, three leading research teams at the forefront of this field have provided their review articles to elaborate on current research in this field.

Dr. Miyazaki and colleagues from Showa University reviewed the current understanding of calpain-associated regulation of endothelial cell (EC) functions [2]. EC function is important for vascular and systemic homeostasis. Accumulating evidence suggests that dysfunction in intracellular proteolytic systems disturbs EC adaptation to the inflammatory environment, leading to the development of vascular disorders (e.g., atherosclerosis and pathological angiogenesis). Moreover, Dr. Miyazaki and colleagues discussed recent findings, including their own, on the contribution of calpain–calpastatin-mediated regulation of EC functions.

In the research performed at Tokushima University, Dr. Nishimoto and colleagues reviewed current knowledge associated with sterile inflammation caused by Toll-like receptor (TLR) 9 and its endogenous ligands in the development of cardiometabolic diseases [3]. Lifestyle-related diseases, such as hyperlipidemia, participate in the pathogenesis of cardiometabolic disorders. However, the underlying mechanisms remain obscure. Dr. Nishimoto and colleagues discussed recent findings, including their own, and suggested that sterile inflammation modulated by TLR9 links lifestyle-related diseases to chronic inflammation in cardiometabolic organs.

Increasing evidence suggests the contribution of chronic inflammation to the pathogenesis of neurodegenerative diseases. Drs. Otani and Shichita from Tokyo Metropolitan Institute of Medical Science summarized recent evidence regarding sterile immune responses in neurodegenerative diseases [4]. Dr. Otani discussed the mechanisms by which sterile inflammation is activated in the central nervous system and introduced recent advances in understanding molecular mechanisms associated with sterile inflammation in several neurodegenerative diseases. Furthermore, an understanding of the detailed molecular and cellular mechanisms in each neurodegenerative disease is necessary for the development of therapeutic strategies.

Sincere gratitude is expressed to the distinguished researchers for their contribution to this thematic series reviews with the hope that these review articles will provide novel insights for the understanding of the mechanism and the development of new therapeutic strategies for cardiovascular–cerebral inflammatory diseases.

## Abbreviations

EC: Endothelial cell
TLR: Toll-like receptor

## References

[1] Virchow R (1989). Cellular pathology. As based upon physiological and pathological histology. Lecture XVI--Atheromatous affection of arteries. 1858. Nutr Rev..

[2] Miyazaki T, Akasu R, Miyazaki A (2020). Calpain proteolytic systems counteract endothelial cell adaptation to inflammatory environments. Inflamm Regen..

[3] Nishimoto S, Fukuda D, Sata M (2020). Emerging roles of Toll-like receptor 9 in cardiometabolic disorders. Inflamm Regen..

[4] Otani K, Shichita T (2020). Cerebral sterile inflammation in neurodegenerative diseases. Inflamm Regen..

